# Chromate Reductase YieF from *Escherichia coli* Enhances Hexavalent Chromium Resistance of Human HepG2 Cells

**DOI:** 10.3390/ijms160611892

**Published:** 2015-05-26

**Authors:** Xuan Liu, Gaofeng Wu, Yanli Zhang, Dan Wu, Xiangkai Li, Pu Liu

**Affiliations:** 1 Department of Developmental Biology, School of Life Sciences, Lanzhou University, Lanzhou 730000, China; E-Mails: kathyliu0505@gmail.com (X.L.); wugf12@lzu.edu.cn (G.W.); zhangyanli12@lzu.edu.cn (Y.Z.); wudan11@lzu.edu.cn (D.W.); 2 Key Laboratory of Cell Activities and Stress Adaptations, School of Life Sciences, Lanzhou University, Lanzhou 730000, China; E-Mail: xkli@lzu.edu.cn

**Keywords:** chromate reduction, *yieF*, HepG2, transfection, Cr(VI) resistance

## Abstract

Hexavalent chromium (Cr(VI)) is a serious environmental pollutant and human toxicant. Mammalian cells are very sensitive to chromate as they lack efficient chromate detoxifying strategy, e.g., chromate-reducing genes that are widely present in prokaryotes. To test whether introduction of prokaryotic chromate-reducing gene into mammalian cells could render higher chromate resistance, an *Escherichia coli* chromate-reducing gene *yieF* was transfected into human HepG2 cells. The expression of *yieF* was measured in stably transfected cells HepG2-YieF by quantitative RT-PCR and found up-regulated by 3.89-fold upon Cr(VI) induction. In chromate-reducing ability test, HepG2-YieF cells that harbored the reductase showed significantly higher reducing ability of Cr(VI) than HepG2 control cells. This result was further supported by the evidence of increased Cr(VI)-removing ability of crude cell extract of HepG2-YieF. Moreover, HepG2-YieF demonstrated 10% higher viability and decreased expression of GSH synthesizing enzymes under Cr(VI) stress. Subcellular localization of YieF was determined by tracing GFP-YieF fusion protein that was detected in both nucleus and cytoplasm by laser confocal microscopy. Altogether, this study successfully demonstrated that the expression of a prokaryotic Cr(VI)-reducing gene *yieF* endowed mammalian cell HepG2 with enhanced chromate resistance, which brought new insight of Cr(VI) detoxification in mammalian cells.

## 1. Introduction

Chromium (Cr) is a heavy metal that is widely used in paints and pigments, leather, metal plating, wood preservation, *etc.* [1,2]. Chromium-contaminated wastes generated from industrial processes and released into the environment have resulted in the contamination of arable land and water. In the environment, Cr ions exist in two common oxidation states. The principal one is Cr(III), the biological role of which became controversial in recent years, although it used to be accepted as an essential element [3]. The other one is Cr(VI), a carcinogen that is highly soluble and easily transported in water [4]. Numerous studies illustrated the toxicity, mutagenicity, and carcinogenicity risk associated with chromate exposure [5,6]. Hence Cr(VI) is more toxic than Cr(III) [7]. Both the International Agency for Research on Cancer and the US Environmental Protection Agency list classified Cr(VI)-containing compounds as Group 1 human carcinogens [8]. Cr(VI) contamination is a serious environmental problem universal, and there are billions of people at threat in the world as they drink water containing carcinogenic quantities of Cr(VI) [9,10].

Due to its structural similarity to SO_4_^2−^, chromate oxyanion can cross the plasma membranes of both bacterial and eukaryotic cells through non-specific phosphate/sulfate anionic transporters [11]. A high concentration of intracellular Cr(VI) can cause oxidative stress, DNA damage, and modulate the activity of regulatory apoptotic gene *p53* [12,13]*.*

The reduction of Cr(VI) in mammalian cells mainly relies on metabolites such as ascorbic acid and low molecular weight thiols including reduced glutathione (GSH) and cysteine [14]. Mammalian cells have also been found to be extremely sensitive to chromium. The 50% lethal dose of Cr(VI) in mammalian cells is as low as 0.15 μg/mL [15], much lower than the hygienic standard for drinking water of China which was 0.05 mg/L for Cr(VI) (GB5749-2006). On the other hand, many microorganisms are highly resistant to Cr(VI), employing different mechanisms including efflux or precipitation of Cr and reduction of Cr(VI) by chromate reductases. Bacteria, such as *Escherichia coli*, *Pseudomonas putita* and *Bacillus subtilis*, can reduce Cr(VI) to Cr(III) and significantly detoxify Cr(VI) [16,17,18]. Correspondent chromate reductases, such as YieF and NfsA in *Escherichia coli* [19,20], ChrR in *Pseudomonas putita* [21] and NfrA in *Bacillus subtilis* [22], have been reported in related studies, whereas no effective reductase has been discovered in mammalian cells.

In this study, to test whether prokaryotic chromate reductase could enhance chromate tolerance of mammalian cells, we cloned gene *yieF*, which conferred chromium resistance in *Escherichia coli* to human hepatocellular carcinoma cell line HepG2. The chromate-resistance capabilities of the stable transfectants were subsequently analyzed. The findings might bring some new insight of heavy metal detoxification in mammalian cells.

## 2. Results and Discussions

The hexavalent chromium compounds can cause various biological damages and strong oxidizing effects in eukaryotes including humans [23,24]. In tobacco plants, Jin *et al.* [25] have successfully transformed the chromate-reducing gene *chrR* into tobacco plants and found *chrR* enhanced the Cr(VI) reduction ability of tobacco leaf disks. It is therefore intriguing to know whether the expression of the Cr(VI)-reducing gene in mammalian cells could similarly reduce the toxic compounds of Cr(VI) and enhance the tolerence to Cr(VI).

### 2.1. Expression of YieF under Cr(VI) Stress

**Figure 1:**
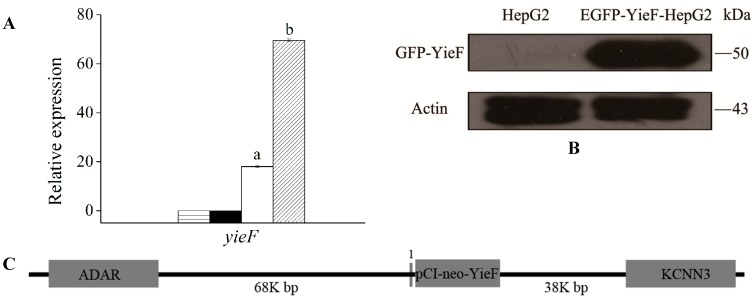
(**A**) The expression levels of gene y*ieF* in different conditions determined by qRT-PCR. Relative level of *yieF* mRNA was measured in HepG2 (▤), Cr(VI)-treated HepG2 (■), HepG2-YieF (▢) and Cr(VI)-treated HepG2-YieF (▨) cells. Data were normalized to *β-actin* expression as a housekeeping gene. The values are the mean of four replicates. There are significant differences (*p* < 0.05) between the two bars marked a and b; (**B**) Detection of GFP-YieF fusion protein by western blotting. Molecular weight of GFP-YieF is about 50 kDa; (**C**) The integration of yieF in human chromosome 1. Grey boxes on the 5ʹ and 3ʹ sides of yieF indicated genes located on Chr 1. Grey line immediately upstream of yieF marked 1 indicate predicted TF binding sites including TBP, Sp1 and CBP100 binding sites. The diagram was not drawn to scale. ADAR, adenosine deaminase, RNA-specific; KCNN3, potassium channel, calcium activated intermediate/small conductance subfamily *N* α, member 3.

Stable HepG2-YieF cells and controls were cultured with or without 5 μM Cr(VI) to determine the relative expression level of *yieF*. The expression of *yieF* was found upregulated by 3.5-fold with Cr(VI) treatment while no induction was observed in control cells (Figure 1A). This result indicated that gene *yieF* was expressed in HepG2 and the expression could be induced by the addition of chromate although the expression vector carrying *yieF* was non-inducible. The presence of YieF protein was confirmed by flow cytometry and western blotting using antibodies against Enhanced Green Fluorescent Protein (EGFP) in EGFP-YieF-HepG2 cells transiently expressing EGFP-YieF fusion protein (Figure 1B). The fluorescence intensity was 22.5%, reflecting relatively low efficiency of transfection (Figure S1).

The interesting finding of inducible expression of *yieF* could be explained by assuming that the genomic region into which *yieF* integrated was responsive to chromate stress. We therefore conducted genome walking to gain some information of the genomic sequence upstream of *yieF* intergration site. The sequencing result of genome walking indicated that *yieF* integrated between 156039118 and 156039119 bp of the plus strand of human chromosome 1 (Figure 1C). The closest annotated gene on the 5ʹ side of yieF was over 60K bp away, which was unlikely responsible for the inducible transcription of *yieF*. Further prediction of transcription factor (TF) binding sites of the immediate upstream region of *yieF* integration site by TRANSFAC 4.0 revealed that -75 to -16 bp region contained binding sites for TBP (TATA box binding protein), Sp1 and CBP100 (CRE Binding Protein 100). As reported, Sp1 and CBP are redox-regulated, therefore herein the binding sites of these TFs might play a role during Cr(VI)-induced oxidative stress to upregulate the expression of *yieF* [26]. Alternatively the chromate stress might elevate the levels of chaperones that could increase the level of functional YieF enzyme.

### 2.2. Enhanced Cr(VI)-Reducing Ability of Cultured HepG2-YieF Cells and Crude Cell Extracts

**Figure 2:**
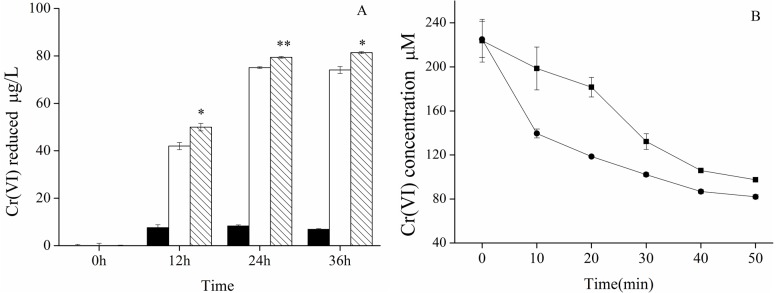

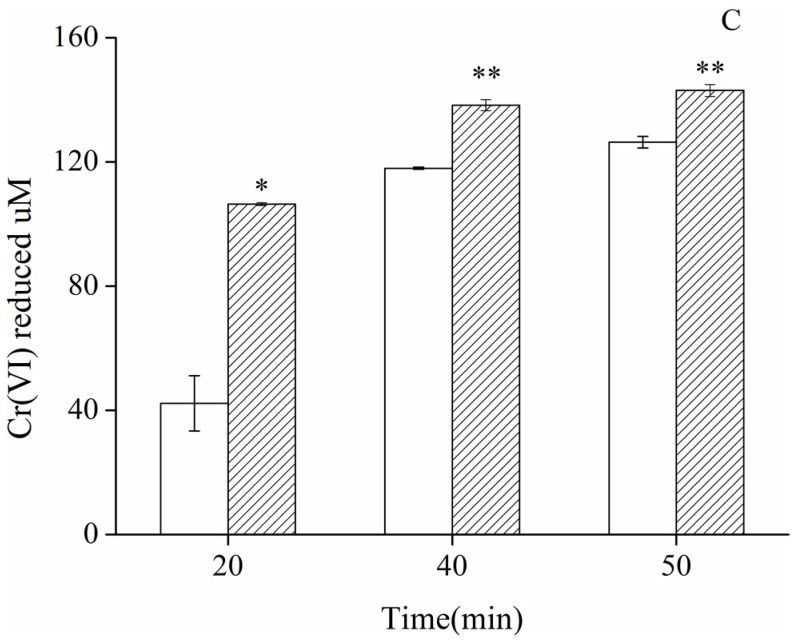
(**A**) Comparison of Cr(VI)-reducing ability of cultured HepG2 (□) and HepG2-YieF (▧) cells at different time points. (■) indicates cell-free control. * *p* < 0.05, ** *p* < 0.01; (**B**) Absolute concentration of remaining Cr(VI) in HepG2 (■) and HepG2-YieF (●) cell crude extract at different timepoints; (**C**) The lessened amount of Cr(VI) in HepG2 (▢) and HepG2-YieF (▨) cell crude extract by calculation. Mean and SEs were obtained from three separate measurements of a representative experiment. Experiments were replicated at least twice. Error bars were in some cases smaller than the size of the symbol. * *p* < 0.05, ** *p* < 0.01.

Both HepG2 cells and HepG2-YieF cells are capable of removing Cr(VI) from culture media within 36 h of culturing with K_2_CrSO_4_, but the reducing ability of the latter was significantly greater than the former (Figure 2A). In addition, to measure the *in vitro* reducing activities of reductants within HepG2-YieF cells, crude cell extracts were prepared from cultured cells. As shown in Figure 2B,C, the reduction rate of HepG2-YieF cell extract was consistently higher than that of HepG2. These results indicated that *yieF* increased the Cr(VI)-reducing ability of HepG2 cells. We also compared the relative cell viabilities of HepG2-YieF and HepG2 and found that the viability of stable HepG2-YieF cells was significantly higher than that of HepG2 cells under Cr stress ( Figure S2).

Although the recombinant YieF enzyme was somewhat effective in enhancing the reducing ability of HepG2, the overall effect and the time window of enzymatic activity appeared limited. Several reasons which deserved further analysis might contribute to the observed result. For example the recombinant enzyme might not be very stable and thus quickly degraded by proteasome. Or the expression might be low due to codon usage bias of mammalian cells. The *yieF* gene was subjected to the Codon Adaptation Index (CAI) measurement and scored 0.69, while a CAI greater than 0.8 was rated as good for expression in mammalian host (Available online: http://www.genscript.com/cgi-bin/tools/rare_codon_analysis). The rare codons in the reductase gene included TTA, GCG, GTA, and *etc.* A new *yieF* gene with optimized codons for mammalian systems could be synthesized and used for expressions in the future. 

### 2.3. Expression Profiles of Glutathione Synthetase and Glutathione Reductase under Cr(VI) Treatment

**Figure 3:**
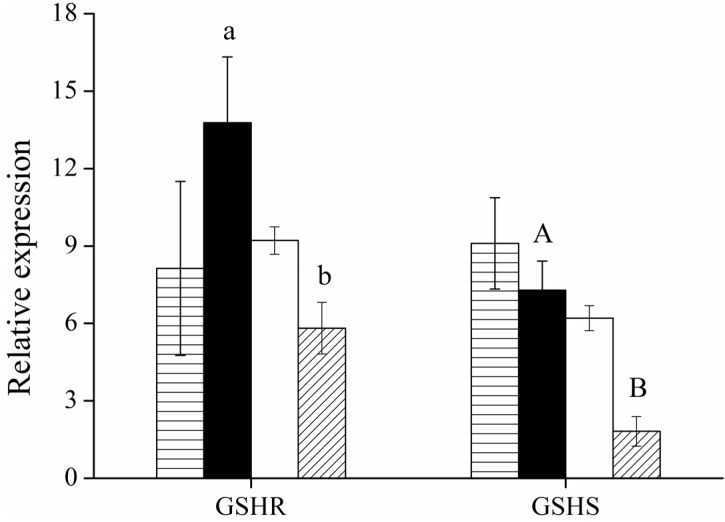
Relative mRNA expressions of glutathione reductase and glutathione synthetase were compared between transfectants and non-transfectants. Bars represent HepG2 without (▤) or with (■) Cr(VI) treatment, and HepG2-YieF without (▢) or with (▨) Cr(VI) treatment. The values are the mean of four individual samples. Data were normalized to the expression of the housekeeping gene *β-actin*. Differences between bar a and b and between A and B are significant (*p* < 0.05).

Some enzymes in mammalian cells are involved indirectly in the reduction of Cr(VI) to Cr(III), for example, glutathione synthetase (for GSH synthesis) and glutathione reductase (for the convertion of reduced GSH from its oxidized form) [27]. The expression of glutathione reductase gene was up-regulated in HepG2 control but down-regulated in HepG2-YieF cells under Cr(VI). Meanwhile, the expression of glutathione synthetase was downregulated in HepG2-YieF cells even without Cr(VI) treatment (Figure 3). These suggested that both of the two GSH-related enzymes were involved in Cr(VI) resistance in HepG2 cells and *yieF* expression might reduce the oxidative stress caused by Cr(VI).

### 2.4. Localization of Recombinant EGFP-YieF Fusion Protein

**Figure 4:**
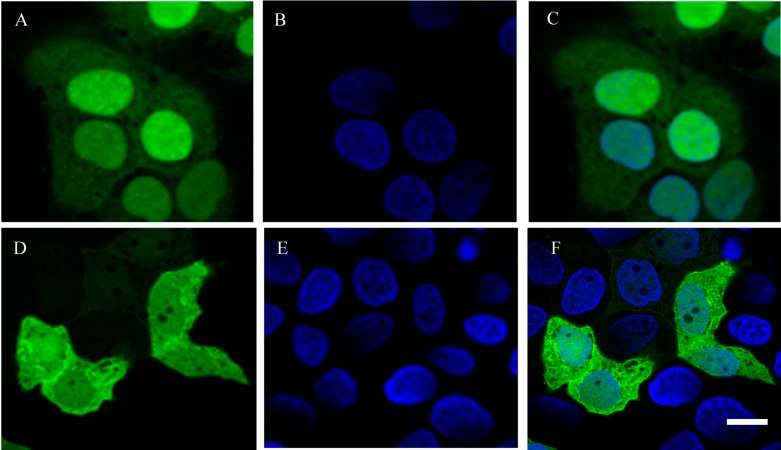
Localization of EGFP and YieF-EGFP fusion protein in transient transfectants. Green fluorescence indicated the localization of empty fluorescent vector EGFP (**A**) and fusion protein YieF-EGFP (**D**). Nuclei were counterstained with Hoechst 33258 (**B**,**E**). **C** is the overlay of **A** and **B**, and **F** is the overlay of **D** and **E**. Scale Bar = 10 μm.

To visualize the localization of YieF, a fusion expression vector carrying YieF-EGFP was constructed and introduced to HepG2. 24 h after transfection, the expression of EGFP and EGFP-YieF was visualized by laser scanning confocal microscopy. The fluorescence was mainly localized in the nucleus of the cells that expressed EGFP only (Figure 4A–C), whereas the fluorescence in YieF-EGFP-transfected cells was detected in both nucleus and cytoplasm (Figure 4D–F), with slightly higher fluorescence intensity in the cytoplasm. This result demonstrated that fusion protein YieF-EGFP was localized in the cytoplasm where Cr reduction took place, possibly to protect the cell nucleus from Cr damage [28]. 

## 3. Experimental Section

### 3.1. Plasmid Construction

The coding region of *yieF* was amplified by PCR from genomic DNA of *Escherichia coli*. The primers used to construct pCI-neo-YieF and pEGFP-N1-YieF plasmid were listed in Table 1. PCR products were digested and inserted into mammalian expression vectors pCI-neo (Promega, Beijing, China) and fusion expression vector pEGFP-N1 (kindly provided by Zhang S.X. lab, Lanzhou, China). Pure Yield plasmid MidiPrep System (Promega, Beijing, China) was used to extract large quantities of plasmids according to the manufacturer’s instructions.

**Table 1:** Primers used in this study.

Primer	Sequences
pCI-neo-YieF	5ʹ-GAATTCTCTAGAATGTCTGAAAAATTGCAGGT-3ʹ/ 5ʹ-GAATTCCCCGGGTCAGATCTTAACTCGCTGAATAA-3ʹ
pEGFP-N1-YieF	5ʹ-GAATTCCTCGAGATGTCTGAAAAATTGCAGGT-3ʹ/ 5ʹ-GAATTCCTGCAGGATCTTAACTCGCTGAATAAACT-3ʹ
*yieF*	5ʹ-AGCTCATTTAATGGCATGG-3ʹ/ 5ʹ-ATCAAGGGAATGTCGGCAA-3ʹ
glutathione synthetase	5ʹ-TGGTCCAGTGCATTTCAGAG-3ʹ/ 5ʹ-TTGGTTCGAAGTAGATGCCC-3ʹ
glutathione reductase	5ʹ-TTACTGCAGTTCCCGGTAGG-3ʹ/ 5ʹ-CTCAGGTCCTTGGTATTCGG-3ʹ
*β-actin*	5ʹ-ACATCCGCAAAGACCTGTATG-3ʹ/ 5ʹ-GCCAG AG-CAGTGATCTCCTT-3ʹ
SP *	5ʹ-TGACGGTTCACTAAACGAGCTCTGC-3ʹ/ 5ʹ-ATCGCAGTTGTTACGACATTTTGGA-3ʹ/ 5ʹ-ATCCCCGTGAGTCAAACCGCTATC-3ʹ

* SP primers were used for Genome Walking to determine the nucleotide sequence upstream of *yieF*.

### 3.2. Cell Culture, DNA Transfection and Cell Selection

HepG2 cells were cultivated in Dulbecco’s minimum essential medium DMEM (HyClone/high glucose) supplemented with 10% fetal calf serum (Sijiqing, Beijing, China), 2 mM l-glutamine, 100 U/mL penicillin, and 100 μg/mL streptomycin at 37 °C and 5% CO_2_. Cells were inoculated into 96-well plate. After incubation for 24 h, the cells were used for transfection. Into each well, 2 μL pEGFP-YieF was added. pEGFP plasmid was used as the control. Transfection was performed using FuGENE^®^6 Transfection Reagent (Promega, Beijing, China) according to the manufacturer’s instructions. Twenty-four hours after transfection, cells were harvested for analysis.

For stable transfection, cells were transfected as described above. Twenty-four hours after transfection, the standard culture medium was replaced by medium containing 800 μg/mL of selection reagent G418 (Geneticin, Invitrogen, Carlsbad, CA, USA) and cells were incubated in it for one week. After that, cells were maintained in 500 μg/mL G418 for two months for further screening. The survived cells were used as stably transfected cells for further analysis.

### 3.3. Western Blotting

For protein extraction, cells were lysed with 100 μL Ripa Lysis Buffer and centrifuged in 13,000 rpm for 10 min. The concentration of protein was determined by bicinchoninic acid (BCA) protein assay. Protein extracts were electrophoretically resolved on 15% SDS-PAGE and transferred onto a nitrocellulose membrane. Western blotting was carried out with standard protocol. Mouse monoclonal antibodies against GFP and β-actin were used as primary antibody (Roche, ZSGB-BIO, Beijing, China). Fluorescein-Conjugated AffiniPure Goat Anti-Mouse IgG was used as secondary antibody (ZSGB-BIO, China).

### 3.4. Genome Walking

HepG2 cells and HepG2-YieF cells were cultivated for 24 h. DNA was extracted with TaKaRa MiniBEST Universal Genomic DNA Extraction Kit Ver.5.0 (TaKaRa, Dalian, China). Then the genomic sequence upstream of the *yieF* expression vector was obtained with Genome Walking Kit (TaKaRa, Dalian, China) according to the manufacturer’s instruction. The PCR products were sequenced by Beijing Genomics Institute (Beijing, China). The primer used for Genome Walking were listed in Table 1.

### 3.5. Chromate Reduction of Cell Culture and Crude Cell Extracts

Cells were grown with 5 μM Cr(VI). One milliliter of culture medium was collected and briefly centrifuged after incubation with Cr(VI) for 0, 12, 24 and 36 h. The concentration of Cr(VI) in the culture medium was determined by DPC method. All experiments were performed with three replicates.

Crude cell extracts were obtained from HepG2 cells and HepG2-YieF cells. 2 × 10^7^ cells were collected and disrupted by ultrasonication in ice water, and then centrifuged in 12,000× *g* for five minutes. The supernatants were mixed with 200 μM K_2_CrSO_4_ solution and incubated in 37 °C water bath. One millilitre sample was taken out at 10, 20, 30, 40, and 50 min after incubation. The Cr(VI) concentration of the samples were determined by DPC method [29]. All experiments were performed in three replicates.

### 3.6. Expression Profiles of YieF, Glutathione Synthetase and Glutathione Reductase under Cr(VI) Treatment

HepG2 cells and HepG2-YieF cells was cultivated with or without 5 μM Cr(VI) for 24 h. Then cells were harvested and total RNA was extracted using RNAiso plus (Takara, Dalian, China) including a DNase digestion step. PrimeScript^®^RT reagent Kit (Takara, Dalian, China) was used for reverse transcription to generate cDNA.

Quantitative real-time PCR was carried out by SYBR Premix ExTapTM II (TaKaRa, Dalian, China) in a total volume of 10 μL in Mx3005P QPCR Systems (Agilent, Santa Clara, CA, USA). The program of PCR was as followed: one cycle of 95 °C for 30 s; 40 cycles of 95 °C for 5 s, 58 °C for 30 s. For each PCR, a no-template reaction was included as negative control. Each cDNA sample was tested in duplicate. Gene *β-actin* was used as a reference gene. All primers were in Table 1. The following analysis was done with MxPro-Mx3005P software.

### 3.7. Laser Scanning Confocal Microscopy

The transiently transfected cells were grown on coverslips and fixed with 4% paraformaldehyde solution for thirty minutes at room temperature. After fixation, cells were washed and stained with Hoechst 33258 (Sigma, St. Louis, MO, USA). Coverslips were mounted on slides with glycerinum. Images were taken with 100-fold oil objective on Olympus FV1000 fluorescence microscope (Olympus, Tokyo, Japan), and processed using Olympus Fluoview ver.2.1c viewer Software.

## 4. Conclusions

Bacterial chromate-reducing gene *yieF* was successfully transfected and expressed in human HepG2 cells. The tolerence to Cr(VI) and the reduction ability of HepG2 were enhanced. Therefore, transgenic expression of bacterial chromate reductase is potentially useful to increase the resistance to chromate in mammals.

## Supplementary Materials

Supplementary materials can be found at: http://www.mdpi.com/1422-0067/16/06/11892/s1.
