# Supplementary material for: Chromate Reductase YieF from Escherichia coli Enhances Hexavalent Chromium Resistance of Human HepG2 Cells
Source: Int J Mol Sci. 2015 May 26;16(6):11892–902. doi: 10.3390/ijms160611892 (PMC4490421; doi:10.3390/ijms160611892)
Supplement: Supplementary file 1 [file ijms-16-11892-s001.pdf]

Supplementary Information

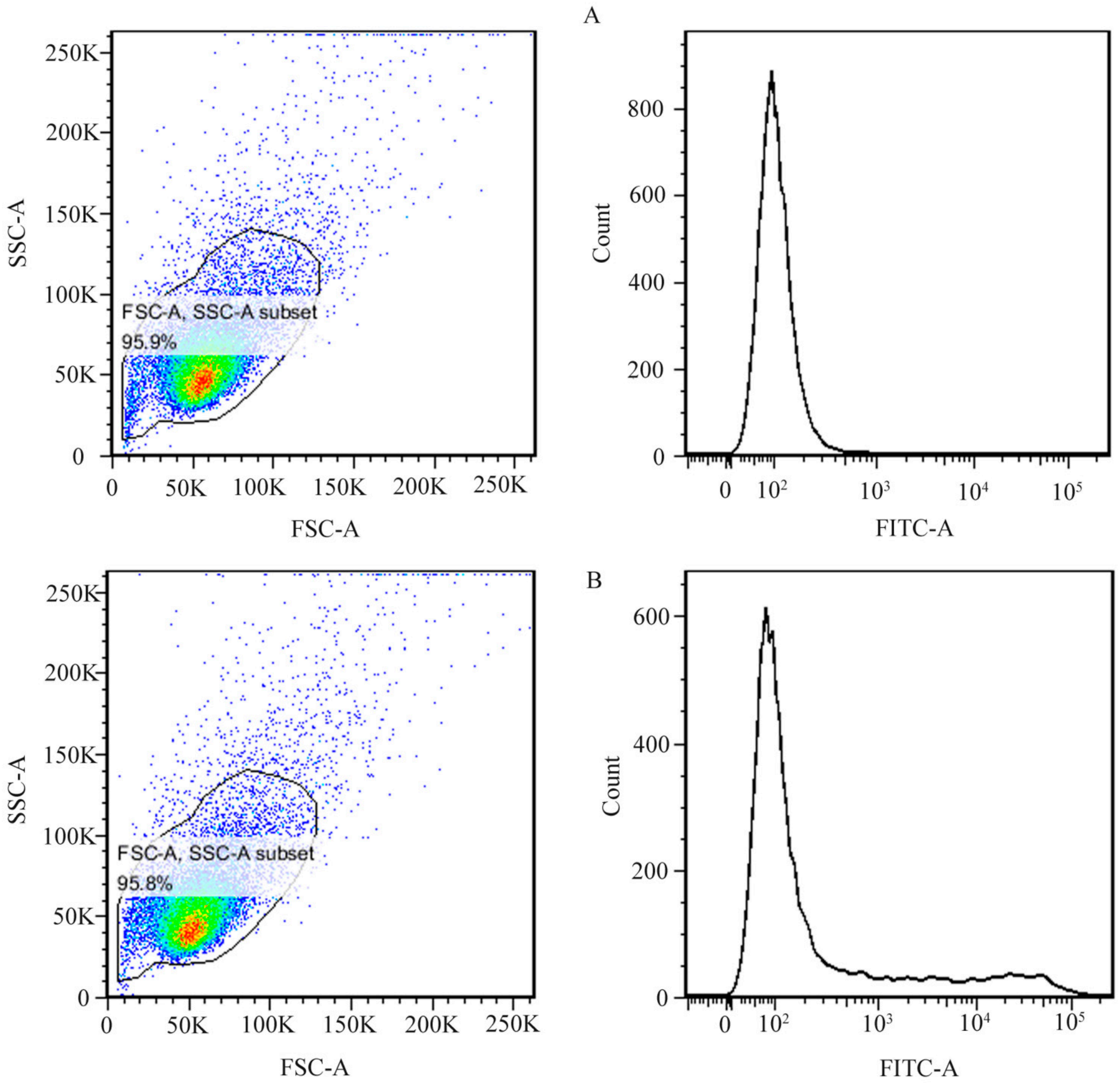

Figure S1. The fluorescence intensity level in (**A**) HepG2 cells and (**B**) EGFP-YieF-HepG2 cells by flow cytometry. The fluorescence intensity was 22.5% in EGFP-YieF-HepG2 cells.

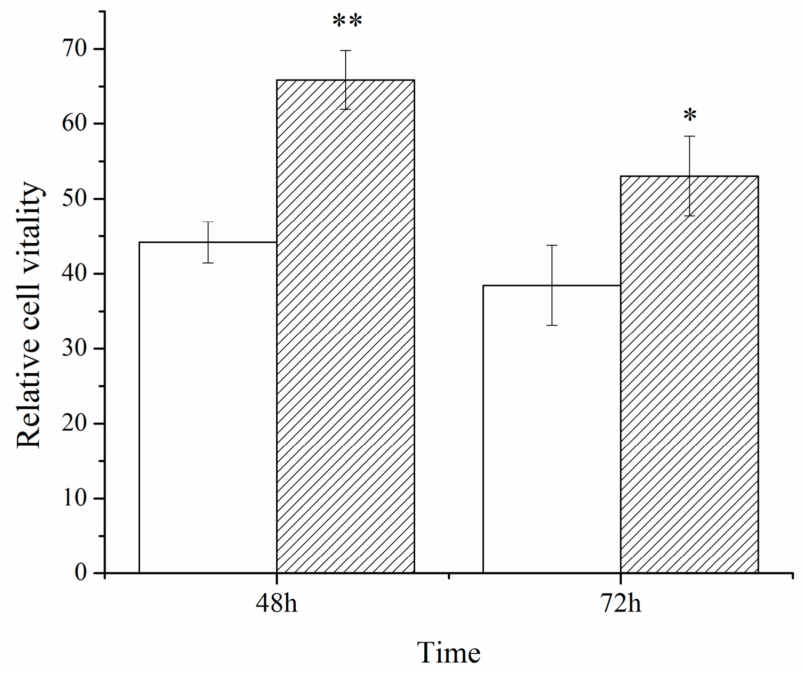

Figure S2. Influence of *yieF* expression on the viabilities of untransfected HepG2 and stable transfectants HepG2-YieF after incubation with 5 μM Cr(VI) for 48 to 72 h. The viability of HepG2 cells grown without Cr(VI) after 48 and 72 h was set as viability level of 100. Bars represent the relative metabolic rates of HepG2 cells (□) and HepG2-YieF cells (▨). * $p < 0.05$,** $p < 0.01$.

Supplementary Materials and Methods:

Flow cytometry to examine the fluorescence intensity

EGFP-YieF HepG2 cells and HepG2 cells were cultivated for 24 h. Then cells were harvested and resuspended in PBS buffer. The fluorescence intensity was determined by FCM using standard protocol.

Cell viability assay

5×10^3 cells were grown in a 96-well plate with or without 5 μM Cr(VI). After incubation for 48 and 72 h, 20 μL of 5 mg/mL 3-4,5-dimethylthiahiazol-3,5-di-phenytetrazoliumromide (MTT) was added into each well. Cells were incubated for 4 h at 37 °C, and then culture medium was replaced by 150 μL DMSO (Sigma). The absorbance of the formazan product at 490 nm was measured on a plate reader [30]. All experiments were performed in three replicates.
